# A Systematic Review of Frailty Interventions in Community-Based Low and Middle-Income Settings

**DOI:** 10.3389/ijph.2025.1608089

**Published:** 2025-04-16

**Authors:** F. Kongngern, M. Prina, S. C. Akhter-Khan, Q. Gao, M. Prince, R. Mayston

**Affiliations:** ^1^ Department of Global Health and Social Medicine, Faculty of Social Science and Public Policy, King’s College London, London, United Kingdom; ^2^ Population Health Sciences Institute, Faculty of Medical Sciences, Newcastle University, Newcastle Upon Tyne, United Kingdom; ^3^ School of Public Health, Imperial College London, London, United Kingdom; ^4^ Population Health Sciences, King’s College London, London, United Kingdom

**Keywords:** systematic review, older people, frail, prefrail, frailty intervention

## Abstract

**Objective:**

To synthesise evidence on the effectiveness of frailty interventions among prefrail and frail community-dwelling older adults living in low and middle-income countries (LMICs).

**Methods:**

The four electronic databases, Embase, Ovid MEDLINE, Global Health, and APA PsycINFO, were searched until 25 September 2023. This review’s protocol was registered on PROSPERO (CRD42022309998). There was no publication time or language restriction. Inclusion criteria included randomised controlled trials and other intervention types of frailty interventions that focussed on community-dwelling older adults (mean age of at least 60 years) with prefrail or frail living in LMICs. The meta-analysis could not be conducted because of the heterogeneity of frailty interventions.

**Results:**

This systematic review included fifteen studies: eleven were single-domain interventions (exercise, nutritional supplementation, and nurse home visits), and four were multidomain interventions (exercise plus nutrition and exercise plus mindfulness). Some evidence from high-quality studies showed that physical exercise interventions successfully addressed frailty.

**Conclusion:**

This systematic review highlights the scarcity of evidence on frailty interventions in LMICs, making it difficult to evaluate their effectiveness. Additional research is needed to focus on specific types of interventions.

## Introduction

The World Health Organization (WHO) predicts that by 2030, there will be 1.7 billion older people aged over 60, and this will increase to 2 billion by 2050, with an annual growth rate of 3%. As populations age, the prevalence of multiple chronic conditions also rises [1]. Health systems in low- and middle-income countries (LMICs) are poor-prepared for these changes, as aging is occurring rapidly in these regions, with resources stretched between large youthful populations and a growing elderly minority [2].

Frailty, a common syndrome in older adults, is associated with age-related physiological changes, reduced body reserves, and diminished ability to cope with stressors [3]. Pre-frailty represents the early stages of these changes [4]. According to a systematic review of 62 countries, the prevalence of frailty and prefrailty were 12% and 46% using physical frailty measures [5]. Whilst there is a deficit in terms of research, far from being a condition present only in wealthy countries, evidence suggests frailty is common among older people in resource-poor settings: according to another systematic review, the pooled prevalence of frailty in LMICs was 17.4%, and prefrailty was 49.3%, which is higher than in HICs [6].

The most common clinical presentations of frailty include fatigue, unintentional weight loss, recurrent infections, falls due to impaired gait and balance, and spontaneous falls in severe cases [7]. Factors contributing to frailty include biological elements such as sarcopenia, malnutrition, inflammation, and infection, as well as psychosocial factors like poverty and social isolation [8].

In clinical settings, frailty is typically diagnosed using either Fried’s Frailty Phenotype, which includes five criteria (weak grip strength, slow gait, unintentional weight loss, exhaustion, and low physical activity), or the Frailty Index (FI), which includes 30 or more signs, symptoms, co-morbidities, and laboratory findings [8]. Frailty is dynamic, with individuals moving between robust/non-frailty, pre-frailty, and frailty states [6]. Those meeting one or two of Fried’s criteria are considered pre-frail, while those scoring between 0.25 and 0.8 on the Rockwood’s Accumulation of Deficits Model or Frailty Index (FI) are classified as pre-frail [9].

Frailty has significant consequences. Physically, it is linked to chronic diseases, complications like falls and fractures, hospitalisations, and disability [7]. A meta-analysis by Fan et al. [10] found frailty increased mortality risk: the pooled risk ratio of all-cause mortality was 2.41 (95% CI 2.07–2.80) for frail women and 2.94 (95% CI 2.12–4.09) for frail men (as defined by using the frailty phenotype) [10]. Muscle mass loss can lead to reduced mobility, dependency, and limitations in daily life [11]. Additionally, frailty is associated with mental health issues such as depression, anxiety, and social isolation [12]. The broader societal consequences of frailty include increased healthcare costs and financial strain on families and communities [13].

Several systematic reviews from HICs suggest that frailty and pre-frailty can be reversed through interventions targeting different aspects of the syndrome. For example, multi-component exercise programs that include resistance, balance, and flexibility exercises have proven effective in improving health outcomes for frail older adults [14, 15]. These programs are also beneficial in improving cognitive function. Multidomain programs combining exercise, nutrition, and other interventions, such as health education and home visits, are particularly effective [16, 17]. A meta-analysis by Macdonald et al. [18] found that resistance-based exercise, particularly when combined with nutritional supplementation, is the most effective strategy for preserving functional capacity in ageing adults [18].

Despite the growing number of older adults in LMICs and the increasing prevalence of frailty, there is a need for more studies, and systematic reviews focused on frailty interventions in these countries. Findings from HICs may is not directly applicable to LMICs due to differences in healthcare systems, cultural perceptions, and resource availability. For instance, access to protein-rich foods and the acceptability of physical activity programs may vary significantly across different regions [19]. Healthcare systems in LMICs are often under-resourced, with limited clinical guidelines for chronic diseases, few specialists in geriatrics, and poorly trained healthcare workers in primary care [20]. Economic pressures and social perceptions about ageing can further discourage older adults from seeking medical help [21, 22].

There is a need to synthesise evidence from LMICs to identify effective frailty interventions in these settings. Interventions for older adults living in community rather than institutional settings are a particular priority, given that residential long-term care remains rare: the vast majority of older prefrail and frail older adults in LMIC live in communities, supported by family members. Research is required to determine recommended interventions’ feasibility, access, and acceptability within resource-limited environments, including dietary changes and exercise programs. Healthcare providers, policymakers, and advocates need relevant evidence to rapidly develop targeted interventions and services to address the unmet needs of older populations in these regions [23].

## Methods

### Search Strategy

**FIGURE 1:**
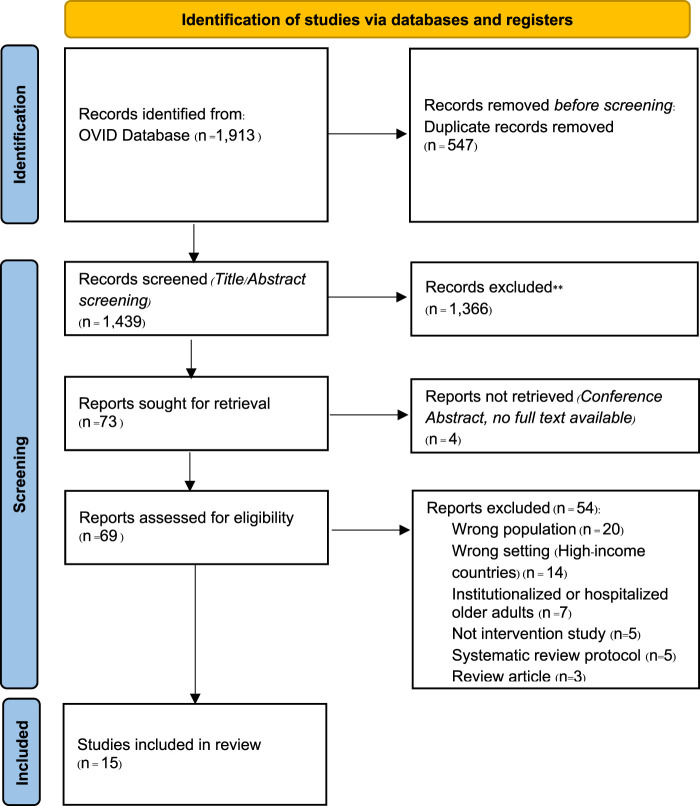
The Preferred Reporting Items for Systematic reviews and Meta-Analyses (PRISMA) Diagram (London, United Kingdom. 2024).

The systematic review protocol was registered on PROSPERO (CRD42022309998). The Preferred Reporting Items for a Systematic Review and Meta-Analysis (PRISMA) guidelines were followed, as shown in Figure 1. Literature searches were conducted using OVID, which includes Embase, Ovid MEDLINE, Global Health, and APA PsycINFO. Search terms included (frail* OR prefrail*) AND (intervention* OR physical exercise OR physical activity OR nutri* OR nutri* supplement* OR brain exercise OR brain stimulation OR cognitive stimulation OR randomised controlled trial* OR random allocation OR double blind method OR single blind method OR clinical trial* OR controlled clinical trial* OR multicenter stud* OR experimental stud*) AND the list of LMICs (per World Bank criteria). The search covered literature up to September 25, 2023.

### Inclusion and Exclusion Criteria

Inclusion criteria were: 1) full-text peer-reviewed publications, 2) randomised controlled trials or other intervention studies (e.g., quasi-experimental, pre-post test studies, and non-randomised controlled trials) conducted in LMICs, 3) community-based interventions delivered in primary care settings, outpatient clinics, homes, or other community locations (e.g., day centres, churches), 4) participants identified as prefrail or frail using any frailty screening tool, 5) community-dwelling older adults in LMICs with a mean age of at least 60. Exclusions were hospitalised or institutionalised individuals, including those living in nursing homes and those with terminal illnesses. There were no language or time restrictions. Deduplicated references from OVID were screened using Rayyan.

### Data Extraction and Quality Assessment

Two reviewers (FK and SA) independently screened titles and abstracts, followed by full-text assessments for eligibility. Disagreements were resolved through discussion. A third reviewer (RM) was available to resolve any disagreements. Extracted data included author(s), year, study design, participants (number and gender), frailty criteria, study inclusion criteria, intervention characteristics (type, content, frequency), outcomes, findings, statistical significance (P-values), and risk of bias assessment. Primary outcomes were frailty status/prevalence. The secondary outcomes were characteristics associated with different aspects of frailty: muscle strength, gait speed, mobility, balance, falls, functional ability, mood, cognitive function, and quality of life.

The risk of bias was assessed by two reviewers (FK and QA) using the Cochrane Risk of Bias tool (RoB 2) for randomised controlled trials and the Risk of Bias In Non-randomized Studies-of Interventions (ROBINS-I) for non-randomized controlled trials. Any disagreements were solved through discussion.

### Data Synthesis

Narrative synthesis was conducted on study characteristics, participants, interventions, and outcomes. Meta-analysis was not performed due to intervention and outcome heterogeneity.

## Results

### Study Characteristics and Participants

Table 1 summarises included studies’ characteristics. Studies were published in peer-reviewed journals between 2013 and 2022. We included fifteen studies in this review. Studies were conducted in 7 countries: China (n = 7), Malaysia (n = 2), Thailand (n = 1), India (n = 1), Indonesia (n = 1), Brazil (n = 2), and Mexico (n = 1). Eleven studies were randomised controlled trials (RCTs). Two were pre- and post-test designs. The other two studies were non-randomised controlled trials and quasi-experimental studies. The sample size ranged from 17 to 203. The total of participants included in all studies was 1,335. Most studies (n = 12) used Fried’s criteria to define frailty. Seven studies recruited prefrailty, while four recruited frailty or cognitive frailty. Four studies enrolled both prefrailty and frailty.

**TABLE 1:** Characteristics of the included studies (London, United Kingdom. 2024).

Author, year	Year of study	Countries	Study designs	Using criteria for frailty screening	Inclusion criteria	Intervention
Badrasawi et al. [24]	23 Mar to 14 Jul, 2014	Malaysia	RCT	Fried’s criteria	Prefrail, ≥60	Nutrition: L-carnitine
Chittrakul et al. [25]	N/A	Thailand	RCT	Fried’s criteria	Prefrail, ≥65, PPA composite risk of falling ≥0	Exercise: Multi-System Physical (MPE) Exercise
Dun et al. [26]	14 Sep 2020 to 31 May 2021	China	RCT	Fried’s criteria	prefrail, ≥65	Exercise: Xiangya Hospital circuit training (X-CircuiT)
Favela et al. [27]	Between Feb and Mar 2009	Mexico	RCT	Frailty Index (FI) (Recruitment process)	≥60, FI ≥ 0.14	Other: Nurse Home Visit
Jiayuan et al. [28]	N/A	China	RCT	Fried’s criteria (Outcome measurement)	Prefrail and frail, ≥65, CDR 0.5	Multidomain: mindfulness and exercise (Tai Chi Chuan)
Lai et al. [29]	Apr 2020 and Sep 2020	China	RCT	Fried’s criteria	Prefrail, ≥60 KPS >80	Exercise: lower limb resistance exercise
Lin et al. [30]	Recruitment between Dec 2018 to Jun 2019	China	RCT	Fried’s criteria	cognitive frailty, ≥60	Exercise: Baduanjin
Neto et al. [31]	Jan to Aug 2010	Brazil	RCT	Edmonton Frail Scale (EFS)	Prefrail, ≥60–75, fulfill handgrip strength by Fried’s criteria	Other: synbiotic intake
Roschel et al. [32]	Oct 2015 and Aug 2017	Brazil	RCT	Fried’s criteria	Prefrail and frail, ≥65	Multidomain: exercise (resistance training programme) and nutrition (supplement-based nutritional strategies)
Wan et al. [33]	N/A	China	RCT	Fried’s criteria	cognitive frailty, ≥60	Exercise: Baduanjin
Wang et al. [34]	1st Mar to 30th Sep 2019	China	RCT	Edmonton Frail Scale (EFS)	frail, ≥65	Exercise: Baduanjin, strength training, and endurance training
Adnan et al. [35]	N/A	Malaysia	Quasi experimental study	Fried’s criteria	Prefrail, ≥60	Exercise: Community-Based Muscle Strengthening Exercise (COME) programme
Chatterjee et al. [36]	Oct 2012 to Dec 2014	India	Pre-test and post-test study	Fried’s criteria	Prefrail, ≥60	Multidomain: exercise (Nordic Walking; NW) and nutrition (Individualised Nutritional Supplementation; INS)
Kang et al. [37]	30 Aug 2017, to 30 Nov 2017 (recruitment)	China	Non-RCT	Fried’s criteria	Prefrail and frail, ≥60	Multidomain: exercise (home-based resistance exercise programs) and nutrition (whey protein)
Riviati et al. [38]	N/A	Indonesia	Pre-test and post-test study	Fried’s criteria	Frail, ≥60	Nutrition: Omega-3

### Intervention Characteristic

Table 2 summarises the intervention characteristics. There were eleven single domain intervention studies. Three intervention types were identified: exercise (n = 7), nutritional supplementation (n = 3), and home visit (n = 1). There were four multidomain intervention studies: exercise plus nutrition (n = 3) and exercise plus mindfulness (n = 1).

**TABLE 2:** Intervention characteristics and outcomes (London, United Kingdom. 2024).

Author, year	Intervention types	Content	Frequency	Outcome measure	Main findings	Risk of bias assessment^a^
Randomised controlled trials (RCTs)						
Badrasawi et al. [24]	Nutrition: L-carnitine	All participants took the L-carnitine supplementation of one capsule (500 mg)	3 times/day after meals, 10 weeks	1-Frailty assessment; Fried’s criteria, Frailty Index (FI) 2-Physical activity; Physical Activity Scale for Elderly (PASE) 3-Nutritional status and anthropometric measurements; weight, height, mid-upper arm circumferences, waist circumferences, hip circumferences, calf circumferences, fat mass, fat free mass, soft lean mass, skeletal muscle mass, basal metabolic rate 4-Cognitive function; Mini-Mental State Examination (MMSE) 5-Physical function assessment; hand grip test, 30-s chair stand test, 8-ft time up and go test, 2-min step test, gait speed test at rapid pace, (Peak Expiratory Flow Rate (PEFR), and shoulder strength test 6-Basic Activities of Daily Living (BADL) and Instrumental Activities of Daily Living (IADL) 7-Frailty biomarkers; IL-6, TNF-alpha, and IGF-1	1-along with Fried criteria, four subjects (three from the L-carnitine group and one from the control group) changed from prefrail to robust after the intervention - statistically improved Frailty Index score (p < 0.05) 2-no statistical differences in PASE 3-no statistical differences in nutritional status and anthropometric measurements 4-no statistical differences in MMSE 5-statistical differences in hand grip strength (p < 0.05) - no statistical differences in other physical function assessment 6-no statistical differences in ADL and IADL 7-no statistical differences in frailty biomarkers	Some concerns
Chittrakul et al. [25]	Exercise: Multi-System Physical (MPE) Exercise	A Multi-system Physical Exercise (MPE) programme was designed for fall prevention and implemented in the intervention group. It consisted of four parts: proprioception training, muscle strength training, reaction time exercise training with auditory cues, and postural balance training	3 days/week, 12 weeks	Primary outcome: 1-Fall risk; Physiological Profile Assessment (PPA) for fall risk Secondary Outcome: 2-The fear of falling; Thai Fall Efficacy Scale International questionnaire 3-Depression; Thai Geriatric Depression Scale (TGDS) 4-Health-Related Quality of Life (HRQOL); 36-Item Short-Form Health Survey questionnaire (SF-36) Thai version	1-statistically differences in fall risk scores (p < 0.001) 2-statistically significant decrease in fear of falling scores (p < 0.01) 3-statistically differences in decrease in depression scores (p = 0.001) 4-statistically differences in increase in HRQOL (p < 0.05)	Low
Dun et al. [26]	Exercise: Xiangya Hospital circuit training (X-CircuiT)	1. X-CircuiT was scheduled for 3 months as follows: 46 min/session (4.5-min warm-up, 6.5-min aerobic training, 6-min acupoint patting, 15-min elastic resistance training and 14- min flexibility training), three supervised sessions per week, once every other day 2. Control group received 1-time advice on physical activity according to current evidence (150 min or more per week of moderate to vigorous physical activity), without supervised exercise Participants in the X-CircuiT group were gathered in Qing-Yuan community health centre and were taught the movements of X-CircuiT by a clinical exercise physiologist, who then supervised all subsequent X-CircuiT sessions	three sessions/week for 3 months	Primary outcome: 1-the proportion of participants with pre-frailty Secondary outcome: 2-absolute risk reduction (ARR) in pre-frailty and number needed to treat (NNT) 3-senior fitness and body composition 4-clinical assessment parameters	1. The proportion of pre-frailty was significantly lower in the X-CircuiT group than the control (14% versus 95%, P < 0.001) 2. The ARR and NNT were 82% [95% CI, 65–99] and 1 [1,2], respectively 3. X-CircuiT was associated with significant improvements in senior fitness indicators and body composition 4. No significant difference in blood chemistry, carotid ultrasound and echocardiography parameters was found between groups	Some concerns
Favela et al. [27]	Other: Nurse Home Visit	1-Nurse home visits including alert button (NV + AB); Participants received home visits by a nurse. The nurses took medical history and identified patient’s improvement areas, also modifiable of home environment, and talked about changing lifestyles. The nurses suggested their patients and relatives about how to success in lifestyles changes and negotiate the goal with them together. They also reviewed patients’ medical treatment. The patients in this group can press the alert button if they want to contact their nurses. Then the nurses will call back and used the triage protocol application on iPod Touch to solve the problems 2-Nurse home visits only (NV-only); the intervention is the same as above details, but this group did not include the alert button 3. Control group; they only receive the usual care from the Family Medicine Clinic and no intervention applied in this group	once a week, 9 months	1-The prevalence of frailty at the end of follow-up	1-The baseline prevalence of frailty was 61.65%. The adjusted prevalence of frailty at the end in NV + AB group was 23.3% versus 58.3% in the control group	Some concerns
Jiayuan et al. [28]	Multidomain: mindfulness and exercise (Tai Chi Chuan)	- First stage; group intervention for 1 h in senior or community centers - Second stage; individual practice for 1 h The interventions assigned in 3 groups separately in places and time to avoid group contamination 1-Group 1 (Mindfulness); there are four types of meditation (gentle yoga, body scan, walking and sitting meditation). For group session, it started with short review for 10 min to solve the problems, then exercise for 45 min, finished with summary for 5 min 2- Group 2 (Tai Chi Chuan: TCC); The participants were taught TCC postures such as Starting Posture, Cloud Hands, etc. For the group session, it started with warm-up for 10 min (joint movement and muscle stretching), then 45 min of exercises, and cool-down (relaxing and deep breathing) for 5 min. For individual practice, the participants continued practicing under supervision 3- Group 3 (Mindfulness-based Tai Chi Chuan: MTCC); it is the mixed exercises between mindfulness and TCC. For the group session, the participants learnt about mindfulness and TCC connections. They practiced various MTCC forms. The session started with warm-up for 10 min, exercises for 45 min and cool-down for 5 min. The participants continued individual practicing under supervision	1-first stage; twice a week, 3 months 2-s stage; twice a week, 3 months	Primary outcome: 1-The rate of cognitive frailty (CF) Secondary outcome: 2-Cognitive function; MMSE and Physical Function; Short Physical Performance Battery (SPPB), Timed Up and Go test (TUG), The 30-s Chair test	1-statistically differences in the rate of CF (p < 0.05) 2-statistically differences in cognitive function and physical performance improvement (p < 0.05) 3-lower prevalence of frailty and better cognitive function and physical performance in Group 3 (p < 0.05)	Low
Lai et al. [29]	Exercise: lower limb resistance exercise	There are three steps of the lower limb resistance exercise design by the rehabilitation physicians for this study which consisted of warm-up, training, and relaxation. These exercises aim for strengthen lower limb muscle group, including the quadriceps femoris, gluteus maximus, and gastrocnemius muscles (e.g., knee bending, knee extension, tiptoes standing, etc.)	three times/week, 12 weeks	1-Quadriceps femoris muscle strength (kg) 2–6-min walking test (m) 3–30-s sit-to-stand test (times) 4–8-ft “up and go” test (s) 5-Daily activity energy expenditure (kcal) 6-Metabolic equivalent	statistically differences in all outcomes (All p < 0.05)	Some concerns
Lin et al. [30]	Exercise: Baduanjin	- Baduanjin exercise training group (BEG) received both a 24-week Baduanjin exercise training and the same health education program as the control group. 60 min per day, 3 days per week, consisting of a 15-min warm-up, 40 min of Baduanjin training, and 5 min cooldown - control group (CG) received only a health education program on nutrition and diet- related knowledge, which consisted of lectures (30 min per session, once every 8 weeks)	three times a week for 24 weeks	1. Cerebral blood flow 2. Cognitive ability was assessed using Beijing Version of the Montreal Cognitive Assessment (MoCA) 3. Physical frailty was assessed using the Chinese version of the Edmonton Frailty Scale (EFS)	1. After the intervention, PSV and MBFV in RMCA, PSV in LMCA, and PSV, MBFV and EDV in BA were significantly different between the two groups (all P < 0.05) 2. The average MoCA scores were increased 2.51 (SD, 0.32) in the BEG, and significantly higher than in the CG (0.34 ± 0.44; P < 0.001) 3. The average EFS scores were decreased 1.94 scores (SE, 0.20) in the BEG, significantly lower than in the CG (-1.16 ± 0.23; P = 0.012)	Low
Neto et al. [31]	Other: synbiotic intake	Intervention group got one daily dose of synbiotic substance, registered by the Brazilian Agency of Sanitary Surveillance-ANVISA (6 g Frutooligossacarides, 108–109 CFU *Lactobacillus* paracasei, 108–109 CFU *Lactobacillus* rhamnosus, 108–109 CFU *Lactobacillus* acidophilus and 108–109 CFU Bifidobacterium lactis)	once a day, 3 months	1-Grip strength 2-Body mass index 3-Skin fold measurements 4-Waist, hip, and calf circumferences 5-Bioelectrical impedance analysis (BIA) 6-Blood samples for IL-6 and TNF-α	No statistically differences between groups in all outcomes	Low
Roschel et al. [32]	Multidomain: exercise (resistance training programme) and nutrition (supplement-based nutritional strategies)	1-All participants undertook resistance training programme. Training sessions comprised with five workouts for the major muscle groups (inclined leg press, leg extension, horizontal bench press, shoulder press, and lat pull down). Training load increased every 4 weeks and ranged from 2 sets at 50% 1-RM (training for first 4 weeks) to four sets at 70% 1-RM (training for last 4 weeks) for each exercise 2-Supplementation - Sub-investigation 1; participants took 3 × 2.5 g/d of either leucine or placebo (alanine) after breakfast, lunch, and dinner - Sub-investigation 2; participants were given 2 × 15 g/d of either soy, whey, or placebo (corn starch) after breakfast and dinner - Sub-investigation 3; participants received creatine (2 × 3 g/d) in combination with either whey (2 × 15 g/d) or placebo (corn starch) (2 × 15 g/d), whereas an additional group were given only placebo (2 × 15 g/d) - Sub-investigation 4; male and female participants were given either whey or placebo following the same pattern as above. Participants mixed the supplements in 150 mL of water	1-exercise: twice a week, 16 weeks 2-supplementation: details as in content, 16 weeks	1-Muscle function; a battery of physical tests including upper- and lower-limb dynamic (1-RM tests) and isometric strength (handgrip and knee extension peak torque) and functional tests (TUG and timed-stands tests) 2-Lean mass; Dual-energy X-ray Absorptiometry (DXA) 3-Muscle cross- sectional area 4-Health-related quality of life 5-Bone mass 6-Fat mass 7-Biochemical markers; 25-hydroxyvitamin D, serum calcium, parathyroid hormone, alkaline phosphatase, aspartate aminotransferase, alanine aminotransferase, serum and urinary creatinine, serum and urinary urea, microalbuminuria, urinary proteinuria, cholesterol, HDL-cholesterol, LDL-cholesterol, VLDL- cholesterol, triglycerides, creatine kinase, lactate dehydrogenase, uric acid, ammonia, erythrocyte sedimentation rate, cortisol, growth hormone, insulin-like growth factor 1, free testosterone, total testosterone, sex-hormone binding globulin, serum glucose, serum glycated hemoglobin, and serum insulin	- no statistical differences in all outcomes - whey, soy, leucine, or creatine supplementation ineffective to enhance resistance-training outcome	Low
Wan et al. [33]	Exercise: Baduanjin	- Baduanjin group participated in Baduanjin training for 24 weeks, three times a week. Each training session lasted for 60 min, including 15 min of warm-up, 40 min of Baduanjin training, and 5 min of cool down. Health education on nutrition and diet related knowledge for the elderly was conducted every 4 weeks (at least 30 min per session) - Participants in the control group did not receive any specific exercise training except for the same health education on nutrition and diet as the Baduanjin exercise training group. They were asked to maintain their original activity habits	1-BDJ three times a week for 24 weeks	1. Global cognitive function assessed by using the Fuzhou version of the Montreal Cognitive Assessment (MoCA) 2. Memory assessed using the Wechsler Memory Scale-Revised, Chinese version (WMS-RC) 3. Physical frailty assessed by using the Chinese version of the Edmonton frail scale (EFS)	1. A statistically significant increase was found for the Montreal Cognitive Assessment (MoCA) scores (p = 0.002) 2. There was a statistically significant in Memory Quotient (MQ) scores (p = 0.019) for the Baduanjin training group 3. There was a significant decrease for the Edmonton Frailty Scale (EFS) score (p = 0.022) for the Baduanjin training group	High
Wang et al. [34]	Exercise: Baduanjin, strength training, and endurance training	The subjects participated in three separate intervention programs, which comprised endurance training, strength training, and Baduanjin. At the beginning of the intervention, each group spent 20 min warming up with various exercises. The Baduanjin training in the BDJ group lasted for 60 min, whereas the Baduanjin training in the BDJSE group lasted for 30 min and then continued for 30 min of strength and endurance exercise. The SE group completed a strength and endurance workout that lasted for 60 min. Ahead of the end point of the intervention, there was a 10-min cool-down period for all groups	24 weeks	1. 10-m maximum walk speed (10 m MWS) 2. Grip strength 3. The timed up-and-go test (TUG) 4. The 6 min walk test (6 min WT)	1. 10 m MWS was a significant interactive influence between group and time 2. No significance difference in grip strength 3. TUGT, there was a significant interactive influence between group and time 4. No significance difference in 6 min WT	Low
Non-randomised controlled trials (Non-RCTs)					
Adnan et al. [35]	Exercise: Community-Based Muscle Strengthening Exercise (COME) programme	COME (Community-Based Muscle Strengthening Exercise) programme. This includes major muscle strengthening for both upper (e.g., biceps curl, push-ups, etc.) and lower extremities (e.g., toe stands, squats, side hip raise, etc.). All exercises start with a warm-up followed by different sets of exercises every week	twice a week, 12 weeks	1-Timed up and go (TUG) 2-Berg balance scale (BBS) 3-Sit-to-stand 4-hand grip strength	no statistical differences between pre and post across all study outcomes	Moderate
Chatterjee et al. [36]	Multidomain: exercise (Nordic Walking; NW) and nutrition (Individualised Nutritional Supplementation; INS)	1-Group A: Adapted indoor Nordic walking (NW only) training; the participants were trained to walk using two walking poles push against the ground. They maintain their natural gait by engaging their trunks and upper limbs during walking. The participants were advised at the beginning for nutritional improvement but no supplementation) 2-Group B: Individualized Nutritional Supplementation (INS only); the participants received nutritional supplementation based on their individual deficits 3-Group C: NW and INS	For NW 60 min, 3 times/week, 12 weeks	The primary outcome 1-Physical performance, as indicated by Fried’s criteria Other measures include: 2-The nutritional status; Mini Nutritional Assessment (MNA), Nestle Nutrition Institute 3-The concentration of serum albumin 4-Physical domain; Modified Physical Performance Test (MPPT) and Berg Balance Scale (BBS) 5-Function; ADL and IADL 6-Mood; 15-item short form of the geriatric depression scale (GDS) 7-Cognition; Hindi Mental Status Examination (HMSE)	1-statistically differences in gait speed in group A (p = 0.001) and C (p = 0.02) - statistically significant increase in grip strength in group C (p = 0.013) 2-statistically significant improvement in nutritional status assessed by MNA (p < 0.001) 3-statistically differences in albumin concentration between groups A and B (p < 0.001) and group A and C (p < 0.01) 4-statistical differences in MPPT score in group C (p = 0.020) -statistically differences in BBS in group A (p = 0.046) and C (p = 0.024) 5-statistically significant increase in ADL and IADL in all three groups 6-statistically differences in GDS score improvement ingroup B (p = 0.025) and C (p = 0.021) 7-no statistically differences in cognition	Moderate
Kang et al. [37]	Multidomain: exercise (home-based resistance exercise programs) and nutrition (whey protein)	All participants received home-based resistance exercise programs, and participants of the intervention group received daily whey protein supplementation For intervention group, daily whey protein supplementation (32.4 g) was provided. They should have breakfast and lunch or 30 min after resistance exercises in addition to their meals	1-Exercise 30 min, twice a day, 12 weeks 2-Whey protein twice a day, 12 weeks	Primary outcome: 1-handgrip strength 2-gait speed 3-the score of Short Physical Performance Battery (SPPB) Secondary outcome: 4-chair rise test 5-balance score from the SPPB	1-The intervention group significantly improved in handgrip strength p = 0.008 (females); p = 0.007 (males) 2-statistical differences for gait speed (p = 0.003) 3-no statistical differences in SPPB 4-statistical differences for Chair-stand time (p = 0.004) 5-no statistical differences in balance score from SPPB	Moderate
Riviati et al. [38]	Nutrition: Omega-3	Omega-3 was provided 1.2 g once a day	once a day, 12 weeks	1-Muscle strength; Bioelectrical Impedance Analysis (BIA) and muscle dynamometer 2-Physical performance; 6 m walking speed	1-statistically differences in muscle strength; handgrip (p = 0.00) 2-no statistical differences in walking speed	Moderate

^a^ Full details of the risk of bias assessment can be found in Tables 3, 4.

### Exercise

All physical exercise intervention studies were conducted in Asian countries. In terms of intervention delivery: in one Chinese study, lower limb resistance exercises designed by rehabilitation physicians were initiated in rehabilitation centre (and then on ward) and after discharge, patients and families were taught by nurses to continue the exercise at home with 12 weeks of follow-up [29]; professional instructors delivered two interventions; physiotherapists delivered all other interventions. All but one of the interventions were delivered in group format in community halls [29]. Interventions included two multicomponent exercise programmes: multi-system physical exercise (MPE) programme covering proprioception, muscle strengthening, reaction time, postural sway, and lower limb resistance exercises-conducted in primary care centre in Thailand [25] and multicomponent exercise intervention delivered in urban China consisting of five stages: warm-up, aerobic training, Traditional Chinese Medicine (TCM)-acupoint patting, elastic band resistance, and flexibility training [26]. There were three single component programmes: a community-based muscle strengthening exercises (COME) intervention conducted at a community hall in Malaysia [35]. Two studies conducted in China were based on Baduanjin Exercise, one of China’s most popular mind-body exercises. It consists of eight movements with low-medium intensity characterised by symmetrical body postures and movements, breathing control, a meditative state of mind, and mental focus [30, 33]. The last exercise intervention, called a Hybrid Exercise Programme by Wang et al. [34], is a combination of Baduanjin exercise and strength and endurance training exercise [34].

### Nutrition

Three studies conducted nutritional supplementation interventions. In Malaysia, Badrasawi et al. [24] conducted randomised, double-blind, placebo-controlled clinical trial of L-carnitine supplementation among prefrail older people ≥60 years old [24]. Riviati et al. [38] explored the effect of omega-3 supplementation in frail older people ≥60 years old in Indonesia [38]. Lastly, using a small sample (n = 17), Neto et al. [31] conducted 3-month double-blind RCT to evaluate the effect of synbiotic supplementation on frailty markers and body compositions of prefrail older people 60–75 years old in Brazil: synbiotics combine prebiotic-and probiotic-substances which are hypothesised to confer benefits to frail older adults via improvements to gut health and functioning [31, 39].

### Nurse Home Visit

Favela et al. [27] evaluated the effects of nurse home visit intervention on frailty, conducting an unblinded RCT in Mexico to evaluate the effects of nurse home visits, including alert buttons (NV + AV) in reducing frailty; participants received home visits by a nurse [27]. The nurses took medical history and identified patient’s improvement areas, home environment, and lifestyle changes. The nurses made recommendations on how to make lifestyle changes and devise effective goal. They also reviewed patients’ medical treatment. The patients in this group can press the alert button to contact their nurses. Then, the nurses will call back and use the follow guidelines to resolve the problems.

### Exercise Plus Nutrition

Three multi-domain interventions combined nutritional advice and/or supplementation and exercise. Chatterjee et al. [36] conducted a pre-and post-test study in India, which randomly allocated prefrail older people ≥60 years old into three groups: group A Nordic walking (NW) only, group B individualised nutritional supplementation (INS) only, and group C (NW and INS). Nordic walking engages upper and lower body muscles in a constant and alternating motion, increasing cardiovascular and respiratory demands while enhancing endurance, flexibility, and balance [36]. Kang et al. [37] conducted a case-control study in China to evaluate the effect of whey protein supplements on muscle function and frailty status of prefrail and frail older people ≥60 years old in addition to home-based resistance exercise programme [37]. Roschel et al. [32] conducted a 16-week, double-blind, randomised, placebo-controlled trial in prefrail and frail older people ≥65 [32]. All participants undertook a resistance training programme and were randomised into four sub-groups to compare different types of nutritional supplementation: either leucine or placebo (alanine), either soy, whey or placebo (corn starch), creatine combined with either whey or placebo (corn starch) or only placebo, and either whey or placebo.

### Exercise Plus Mindfulness

One study combined mindfulness and exercise (Tai Chi Chuan). Jiayuan et al. [28] recruited prefrail and frail older people ≥65 years old for a 6-month single-blind, three-arm randomised controlled trial in China to compare the effects of mindfulness intervention only, Tai-Chi Chuan intervention only and mindfulness-based plus Tai-Chi Chuan (MTCC) intervention [28].

### Quality Assessment

Table 3 (ROB2) and Table 4 (ROBINS-I) show the risk of bias assessment. Of the fifteen included studies, eleven were evaluated using the ROB2 for quality assessment and four using the ROBINS-I for quality assessment. Six studies were low-risk, and five had some concerns. From the ROBINS-I assessment, all studies (n = 4) were moderate risk. Therefore, only six studies had a low risk of bias.

**TABLE 3:** Quality assessment using the Cochrane Risk of Bias tool (RoB 2) for randomised controlled trials (London, United Kingdom. 2024).

	D1	D2	D3	D4	D5	Overall
Badrasawi [24]	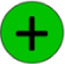	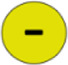	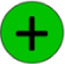	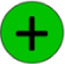	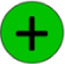	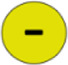
Chittrakul [25]	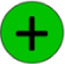	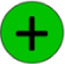	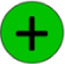	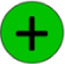	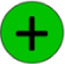	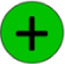
Dun [26]	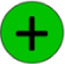	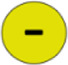	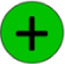	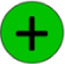	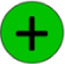	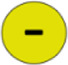
Favela [27]	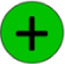	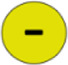	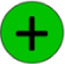	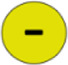	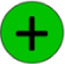	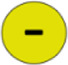
Jiayuan [28]	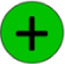	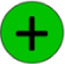	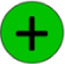	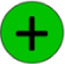	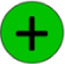	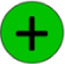
Lai [29]	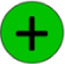	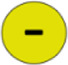	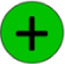	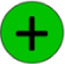	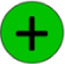	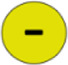
Lin [30]	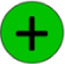	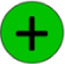	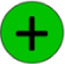	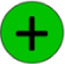	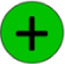	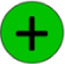
Neto [31]	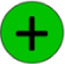	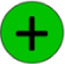	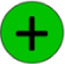	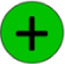	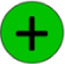	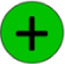
Roschel [32]	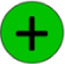	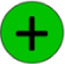	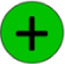	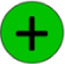	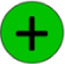	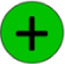
Wan [33]	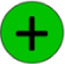	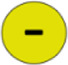	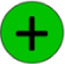	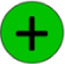	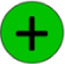	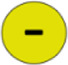
Wang [34]	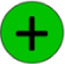	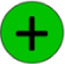	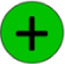	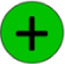	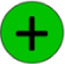	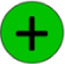

Domain:

D1: bias due to randomization.

D2: bias due to deviations from intended intervention.

D3: bias due to missing data.

D4: bias due to outcome measurement.

D5: bias due to selection of reported result.

Judgement:

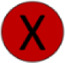
 High.

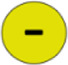
 Some concerns.

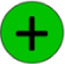
 Low.

**TABLE 4:** Quality assessment using the Risk of Bias In Non-randomized Studies-of Interventions (ROBINS-I) for non-randomized controlled trials (London, United Kingdom. 2024).

	D1	D2	D3	D4	D5	D6	D7	Overall
Adnan [35]	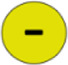	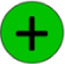	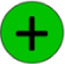	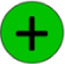	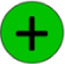	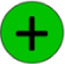	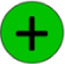	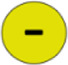
Chatterjee [36]	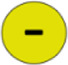	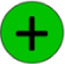	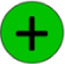	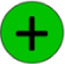	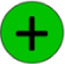	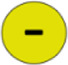	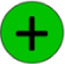	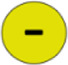
Kang [37]	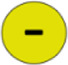	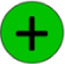	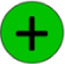	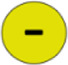	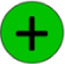	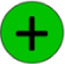	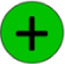	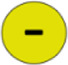
Riviati [38]	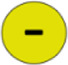	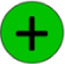	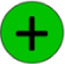	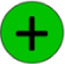	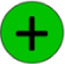	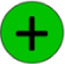	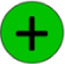	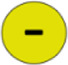

Domain:

D1: bias due to confounding.

D2: bias due to selection of participants.

D3: bias in classification of interventions.

D4: bias due to deviations from intended interventions.

D5: bias due to missing data.

D6: bias in measurement of outcomes.

D7: bias in selection of reported result.

Judgement:

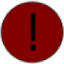
 Critical.

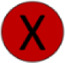
 Serious.

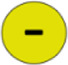
 Moderate.

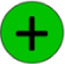
 Low.

### Outcomes

Outcomes are summarised in Table 2.

### Exercise

All but one of the exercise intervention studies reported positive effects on frailty-related variables after 12 weeks of intervention [35]. The COME programme resulted in small positive differences in some frailty-related outcomes: time up and go (TUG) = −0.25 (95%CI –0.89, 0.39), sit-to-stand duration = −0.41 (95%CI –1.17, 0.34), and handgrip strength 0.68 (95%CI –0.92, 2.30). The sit-to-stand duration showed the most significant benefit from the intervention, recording the highest effect size among the outcome measures (0.20). Chittrakul et al. [25] reported decreased risk of fall scores (p < 0.01) as well as decreased fear of falling scores (p < 0.01), depression scores (p = 0.001), and increased Health-Related Quality of Life (HRQOL) (p < 0.05) after 12 weeks of MPE programme [25]. Dun et al. [26] aimed to reverse prefrailty; they found significantly lower proportion of prefrailty in the 3-month X-Circuit intervention group than in the control (14% VS 95%, P < 0.01) [26]. The ARR and NNT were 82% (95% CI, 65–99) and 1 (1-2), respectively. Also, among pre-frail older people, Lai et al. [29] found that 12-week lower limb resistance exercise had a positive effect on mobility measured by 6-min walking test, 30-s sit-to-stand test, 8-ft “up&go” test with statistically significant results (all p < 0.05) [29]. Both studies, which assessed the effects of the 24-week Baduanjin programme, reported that this exercise significantly decreased the Edmonton Frailty Score (p = 0.01 from Lin et al.’s study [30] and p = 0.02 from Wan et al.’s study [33]). A significant increase in Montreal Cognitive Assessment (MoCA) scores (p < 0.001 and p < 0.01respectively) was also found. Another study on 24-week Baduanjin exercise by Wang et al. [34], found statistically significant changes in mobility, measured by a 10-m maximum walk speed (p < 0.01) and timed up and go test (TUGT) (p = 0.04) [34]. However, no differences were found in hand grip strength and the 6-min walking test.

### Nutrition

In 10-week L-carnitine group, frailty index scores and hand grip strength were significantly improved (p < 0.05 for both parameters) [24]. However, there was no difference in frailty biomarkers (interleukin-6 (IL-6), tumour necrosis factor-alpha (TNF-alpha), and insulin-like growth factor-1 (IGF-1), physical function, cognitive function, and nutritional status [24]. Riviati et al. [38] claimed a significant improvement in hand grip strength from omega-3 supplementation for 12 weeks, but the reported data do not support that conclusion, the reported p-value 0.00 is erroreous [38].

The third nutritional study, by Neto et al. [31] found that 3 months of synbiotic supplementation did not benefit body composition or inflammatory cytokines [31].

### Nurse Home Visit

Favela et al. [27] found that Nurse Home Visit for 9 months decreased the prevalence of frailty from 61.65% at baseline to 23.3% (p < 0.05) when an alert button was included (NV + AB) [27].

### Exercise Plus Nutrition

Of the three studies examining exercise combined with nutrition interventions, Chatterjee et al. [36] found statistical differences in gait speed in the NW group (p < 0.01) and NW and INS group (p = 0.02) and statistically significant increase in hand grip strength in the NW and INS group (p = 0.013) after 12 weeks of follow-up. Mood measured by the Geriatric Depression Scale (GDS) was significantly improved in the INS group (p = 0.025) and the NW and INS group (p = 0.021) [36]. There was no significant difference in cognitive status. Kang et al. [37] found that 12-week whey protein supplements improved hand grip strength (p < 0.01), gait speed (p < 0.01) [37], and chair-stand time (<0.01). Roschel et al. [32] found no difference in muscle mass and function according to 16-week nutritional supplementation [32].

### Exercise Plus Mindfulness

Among cognitively frail older people, Jiayuan et al. [28] found lower prevalence of frailty and improvement in physical performance (measured by Short physical performance battery: SPPB and TUGT) and cognitive function (Mini-Mental State Examination: MMSE) among the group receiving mindfulness plus Tai-Chi Chuan intervention (p < 0.05) for 3 months. The intervention also decreased frailty prevalence; 9 participants (30%) reversed to no cognitive frailty [28].

## Discussion

Frailty is a significant global health issue, potentially affecting more older people in LMICs than in HICs. Our systematic review revealed limited evidence on frailty interventions in LMICs, identifying 15 studies, of which only six offered robust evidence (low risk of bias). Methodological concerns in studies with some concerns risk of bias included unblinded study, non-randomisation, or many drop-offs in per-protocol analysis studies. The small number of high-quality studies makes it difficult to draw definitive conclusions.

Unlike findings from HICs, two studies examining nutritional supplementation in LMICs (e.g., synbiotics, whey, creatine, leucine) showed no benefits. Conversely, other reviews from HICs reported modest improvements in physical function and mobility with protein supplementation [40]. According to the systematic review by Yan et al. [41], protein supplementation intervention remarkably improved body weight, muscle mass, muscle strength, and physical performance among frail older adults [41]. Consistent with HICs evidence, our findings suggest physical exercise can improve frailty-related outcomes, including cognitive and physical function, falls, depression, and quality of life [25, 30, 33]. Multi-component exercise programmes (resistance, balance, flexibility) were particularly effective in improving muscle strength [14, 15]. One high-quality LMICs study showed that such interventions reduced fall risk, fear of falling, and depression while improving health-related quality of life [25]. However, more research is needed to understand these effects fully.

Notably, two effective physical activity interventions incorporated mindful movement elements, bridging the mind-body divide often reinforced by biomedicine. These interventions address the psychological aspects of frailty, such as the “frailty identity crisis,” which reflects feelings of despair during the transition to frailty [42]. Psychological challenges, including depression, can worsen frailty and act as barriers to participation in interventions. Research from LMICs highlights that older people often view health decline as a natural part of ageing, face stigma around mental health, and perceive healthcare as inaccessible due to poorly equipped systems, limited old-age specialists, and insufficient insurance coverage.

Interventions addressing psychological, social, and spiritual aspects of frailty are essential. Successful interventions must also consider cultural appropriateness. For example, two reviewed interventions in China incorporated popular cultural practices like Tai Chi and Baduanjin, highlighting the importance of integrating traditional activities. This approach, widely debated in global mental health [43], has yet to become a topic for research in gerontology, where cultural appropriateness is, perhaps, equally important. Given this context, adopting or adapting traditional and/or popular practices as health promotion interventions (where locally relevant ones exist) is a promising line of inquiry in frailty and global ageing research.

Asian governments have invested in health systems and welfare reform to support changing demographics. In China, for example, policy changes target older people living with multimorbidity in terms of increased inputs from the government to the health and social system for older people [23]. In Thailand, National Policies on Ageing exist, such as the National Long-Term Plan for Older Persons [44]. Thailand is prepared for old-age security, including income security, housing and environment, health and healthcare, dependence care, and rights and safety [45].

The demographic and socioeconomic context in LMICs must be addressed. Notably, all the studies we identified were conducted in upper-middle-income countries. All but one of the exercise interventions included in this review were led by physiotherapists or professional instructors (in the case of Baduanjin). This resource is unlikely to be available in low-income countries, where health systems are more stretched and lack specialist health workers and training opportunities. Developing task-shared interventions where non-specialists deliver physical activity programmes could address these gaps. WHO’s Integrated Care for Older People (ICOPE) programme exemplifies this approach. A recent randomised controlled trial of this intervention revealed promising results, with improvements in mobility, vitality and psychological health [46].

### Limitations

We adopted a comprehensive search strategy using four databases. However, it is possible that we missed article due to different language between search terms and language of publication (e.g., search terms were in English, maybe publications were in Thai and could only be found with Thai search terms). The search strategy we adopted has resulted in a small number of studies from which it is difficult to draw strong conclusions. However, this is more likely a finding rather than an artefact *per se* of our approach. The concept of frailty is derived from studies carried out in the West. Using this as a primary search term, we may have missed studies that addressed conditions, states, or symptoms linked to frailty (e.g., depression) but were not labelled as such by researchers. In particular contexts, specific health problems linked to older age may be considered necessary but not covered within the frailty construct. In addition, the validity of frailty measures in other cultural contexts has generally yet to be investigated [47]. Additionally, our last search was conducted in September 2023- it is possible that new studies have been published since then which are not included in this review.

We deliberately focused upon studies carried out among community-dwelling older adults. Even in countries with the largest numbers of older people, residential long-term care, whilst growing rapidly, remains rare and fraught with challenges-including shortages of workers, weak quality regulations [48]: there is a lack of studies carried out in these settings LMICs, despite positive findings from HICs [49]. Rates of frailty among hospitalised older adults in LMICs are high-a recent systematic review estimated the pooled prevalence to be 39% [50]. However, given that the vast majority of older people live in communities and primary healthcare is designed to be accessible to all [51], prioritising the integration of frailty interventions in primary healthcare settings perhaps has the greatest potential for impact.

### Conclusion

Frailty interventions focused on physical activity show promise in addressing frailty among prefrail and frail older adults. However, the small number of high-quality studies and limited sample sizes hinder robust conclusions. Different from the wealth of research in HICs, LMICs face unique cultural, health system, demographic, and socioeconomic challenges, making findings from HICs less transferable.

Future research should investigate intervention feasibility, including delivery methods, implementers, and locations. Community-based delivery may be necessary to address feasibility, cultural appropriateness, and acceptability issues, as well as to maximise impact. Efforts should also focus on raising awareness of older adults’ health challenges as equity issues. Governments must prioritise ageing as a development issue, particularly in regions with rapidly growing older populations.
